# Comparison of Risk Factors and survival of Type 1 and Type II Endometrial Cancers

**DOI:** 10.12669/pjms.324.9265

**Published:** 2016

**Authors:** Tahira Y. Malik, Uzma Chishti, Aliya B. Aziz, Irfan Sheikh

**Affiliations:** 1 Dr. Tahira Yasmeen Malik, Fellow Gynae-oncology, Aga Khan University Hospital, Karachi, Pakistan; 2 Dr. Uzma Chishti, Senior Instructor, Department of Obstetrics and Gynaecology, Aga Khan University Hospital, Karachi, Pakistan; 3 Dr. Aliya B. Aziz, Assistant Professor, Department of Obstetrics and Gynaecology, Aga Khan University Hospital, Karachi, Pakistan; 4 Dr. Irfan Sheikh, Senior Instructor and Statistician, Aga Khan University Hospital, Karachi, Pakistan

**Keywords:** Endometrial cancer, Risk factors, Survival

## Abstract

**Objective:**

To compare risk factors and progression free survival of type 1 & 2 endometrial cancers.

**Methods:**

A retrospective analysis of 149 patients with early stage endometrial carcinoma treated between 1997 and 2012 in Aga Khan University Hospital, Karachi was performed.

**Results:**

A total of 149 patients were analyzed. Type I tumors accounted for 92% of cases in the study while 8% were type II tumors. The mean age, BMI, parity, co-morbidities (hypertension & Diabetes), family history and history of polycystic disease were comparable in both groups. Overall better survival (113 Vs 24 months) was observed for type I endometrial cancer.

**Conclusion:**

Both types of endometrial cancer may share common etiologic factors. Despite the limitation of small numbers in one group this study confirms better survival in type 1 endometrial cancer.

## INTRODUCTION

Carcinoma of corpus uteri is the third common among gynecological cancers in Asians with incidence of 4.3% for all ages and 1.9% mortality among Asians (globocan, 2012). Endometrial carcinoma is the second most lethal gynecologic cancer in the United States, causing over 8,000 deaths annually.^1^ The incidence of Endometrial carcinoma exceeds the incidence of cervical, ovarian, vaginal, and vulvar cancers combined.^2^ Ninety five percent of uterine carcinoma occur in women over 40 years of age usually in the sixth and seventh decades of life. Lifetime risk of developing endometrial carcinoma is 2.5%.^3^ Endometrial cancers have been divided into two types on the basis of histology and clinical outcomes.^4^ Type I endometrial cancers being common and mostly endometrioid adenocarcinomas, are associated with unopposed estrogen stimulation, often preceded by endometrial hyperplasia.

Type II tumors are predominantly serous carcinomas and are commonly estrogen independent, mostly arising in atrophic endometrium and deriving from intraepithelial carcinoma as a precancerous lesion. Type II tumors have poorer prognosis than type I tumors as they are generally poorly differentiated. They only account for 10% to 20% of cases with 40% of death rate among them.^5^ Obesity, metabolic syndrome and external hormone therapy are main risk factors for Type I tumors.^6^,^7^ Others include nulliparity, early menarche, and late menopause, whereas smoking is associated with reduced risk. Risk factors for type II tumors are not well known, mainly because of lack of enough cases to study these less common tumors separately.^8^,^9^

Uterine papillary serous carcinoma and clear cell histology are distinct variants of endometrial cancer with tendency for extra-uterine spread and poor prognosis.^10^ Type 1 endometrial cancers are low risk having better 5 year survival rates whereas, stage-adjusted 5-year survival rates for Type II tumors are significantly worse compared to Type I tumors.^11^

The aim of this study was to compare risk factors and progression free survival of both types of endometrial cancers. Identification of risk factors of both types of cancers would help in devising effective prevention strategies and would significantly elevate health care standards.

## METHODS

After the approval of institutional review board, records of all patients with histology proven endometrial carcinoma, diagnosed and treated at Aga Khan University hospital, Karachi Pakistan from 1997 to 2012, were reviewed retrospectively. All patients with endometroid adenocarcinoma, papillary serous and clear cell carcinoma were included in this study while, patients with uterine sarcomas and mixed mullerian tumors were excluded. Patients who presented with recurrent endometrial carcinoma were also excluded. Patient’s characteristics including age, body mass index, parity, history of smoking, diabetes and hypertension were recorded on pre-designed questionnaire.(Table-I). Histopathological type as endometroid adenocarcinoma and mucinous adenocarcinoma were categorized as type 1 endometrial carcinoma while clear cell, papillary serous carcinoma were categorized as type 2 endometrial carcinoma. History of intake of hormones, family history of cancers, history of radiotherapy and overall survival was obtained from the patients’ records. All of this information was collected on the predesigned questionnaire.

**Table-I:** Patients characeristics

Variables	Type I n=137	Type II n=12	P-Value
Age (Years)	56.66±10.51	61.08±6.69	0.15
BMI	31.85±6.78	32.79±5.66	0.65
Parity	3.75±5.26	4.66±2.93	0.55
Hypertensive	77(56.2%)	9(75%)	0.21
Diabetic Mellitus	55(40.1%)	8(66.7%)	0.07
Family History of Cancers	17(12.4%)	3(25%)	0.20*
Smoking	3(2.2%)	0(0%)	0.61
PCO	2(1.5%)	0(0%)	0.67
HRT	0(0%)	1(8.3%)	0.08
Radiation	47(34.3%)	6(50%)	0.34*
Mortality	5(3.6%)	2(16.7%)	0.10*

Data are presented as mean ± SD and n (%)

* fisher exact test applied

SPSS version 19 was used to analyze the data. Mean and standard deviations for continuous variables and proportions for categorical variables were estimated. Student’s t-test and Fisher exact test were used. Overall survival of the two groups was compared by survival analysis.

## RESULTS

A total of 180 patients with endometrial cancer (EC) were diagnosed and treated at Aga Khan university Hospital between 1997 and 2012. Out of the total, 8 patients had carcinosarcoma, 6 had sarcoma and 17 patients were lost to follow up. Therefore, these 31 patients were excluded and 149 patients were included in this study. One hundred and thirty seven patients had endometroid adenocarcinoma (type I) and 12 patients had papillary serous and clear cell histology (type II). Type I tumors accounted for 92% of cases in the study while 8% were type II tumors. Mean age of the patients was 56.6(SD ±10.51) years in Type I EC group while 61.08(SD±6.69) in type II endometrial cancer group.

Mean BMI of the patients with type I endometrial cancers was 31.85±6.78 while in- patients with type II endometrial cancers was 32.79±5.66 (P value 0.65). Patients in both groups were multiparous with average parity of 3 in type I and 4 in type II group with (P value of 0.55). In type I endometrial cancer patients 77(56.2%) out of 137 were hypertensive while 9(75%) out of 12 patients were hypertensive with P value of 0.21.

Diabetes was present in 55(40.1%) patients with type I endometrial cancer while in type II endometrial cancer group 8(66.7%) were diabetic with P value 0.07. Family history of malignancy was noted in 17(12.4%) patients with type I EC while 3(25%) patients with type II EC were diabetic with P Value of 0.20. Three patients (2.2%) out of 137 patients in type I endometrial group were smokers while no smoking history was noted in type II endometrial cancer group (P value 0.61). History of PCO was positive in two (1.5%) patients with type I EC while no patient had history of PCO in type II EC group (P Value 0.67). No patient had past history of Hormone replacement therapy (HRT) in Type I EC group while 1(8.3%) Patient in type II EC had taken HRT (P Vale 0.08%)

**Fig.1:**
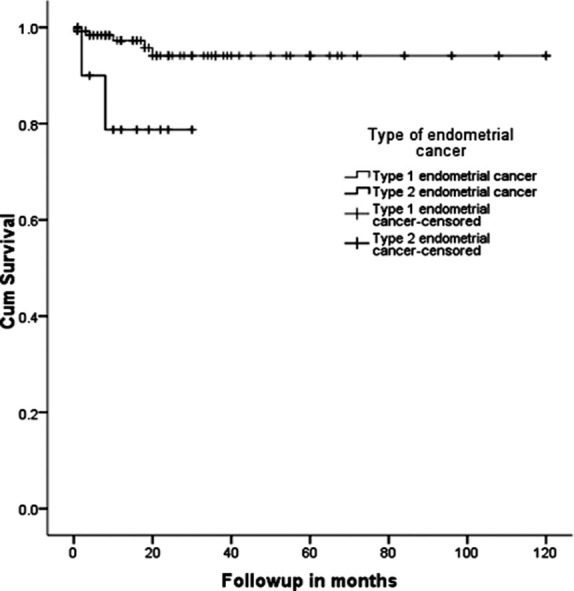
Comparison of overall survival between the two types of endometrial cancer.

Adjuvant radiation was given to 47(34.3%) patients in Type I EC group, while 6(50%) patients in Type II EC group received Radiation (P Value 0.34). 5(3.6%) out of 137 patients with type I EC died of disease while mortality of 2(16.7%) in type II EC group (P value 0.10). Table-II. As shown in survival curve (Fig-1) mean survival time in type I EC patients was 113.68±2.82 months [108.15 to 119.22] (95%CI) while 24.72±3.33 months [18.19 to 31.25] in type II EC patients.

**Table-II:** Survival characteristics.

	Number of cases	Number of event (death)	Number censored	Mean survival time (95%CI)
Type I	137	5	132(96.3%)	113.68±2.82 [108.15 to 119.22]
Type II	12	2	10 (83.3%)	24.72±3.33 [18.19 to 31.25]

## DISCUSSION

Endometrial cancer (EC) is the most common gynecologic malignancy in developed world, with incidence rising in developing countries.^12^ Most women with EC are detected at an early stage and undergo surgico-pathological staging. Endometroid adenocarcinoma (type 1 histology) is most common, seen in 75% to 80% cases and is associated with good prognosis. The 5-year survival rate is approximately 96% in lymph node negative disease and is reduced to 67% in lymph node positive disease.^13^ Only 10% of all endometrial carcinomas are (PSCE) papillary serous carcinomas of the endometrium.^10^

In our study mean age of the patients was 56.6(SD ± 10.51) years in Type I EC group which is consistent with 54 years median age of women in Mahantshetty U et al. study.^14^ Whereas, mean age of patients with type 2 EC was 61.08(SD±6.69) in our study which is not consistent with previous studies, for instance Virginia Benito et al., in which mean age of patients at the moment of diagnosis with type 2 histology was 70(SD±9.34) years (range, 56-93 years; 25%-75%; 63-75). This difference between mean age of type 2 EC in present research and previous researches was probably because of large sample size used in previous researches.^15^

Mean BMI of the patients with type I endometrial cancers was 31.85±6.78 while in patients with type II endometrial cancers was 32.79±5.66 (P value 0.65). Finding of present study reveal no association between BMI and increased risk of endometrial type 1 cancer. BMI difference in type 1 and type 2 endometrial cancers is non-significant. These findings are consistent with results obtained in a previous study conducted by Weiderpass, et al. In which it was found that obesity is unrelated to endometrial cancer risk.^16^ However, findings of present study contradict with general observation which suggests a strong association between type 1 endometrial cancer and BMI. This should be mentioned here that this inconsistency could have arisen because of a small and unmatched sample size. Because of the rare nature of type 2 endometrial cancers, only few cases were included in the study that might have influenced the analysis of data and resulted in misleading findings.

In the present study, incidence of hypertension is high among type 1 and type 2 endometrial cancer groups. In type I endometrial cancer patients 77(56.2%) out of 137 were hypertensive while 9(75%) out of 12 patients were hypertensive with P value of 0.21. However, hypertension could only be considered a risk factor if it is present in obese people. Hypertension could not be taken as risk factor of endometrial cancers independently. Another study that reported similar findings was conducted by Weiderpass, et al. in which it was reported that risk of endometrial cancer was high among those hypertensive individuals who were obese.^16^

Analysis of data reveals that Diabetes was present in 55 (40.1%) patients with type I endometrial cancer while in type II endometrial cancer group 8 (66.7%) were diabetic with P value 0.07. It was found in the present study that diabetes is a risk factor for type 1 endometrial cancer and its incidence is high among type 1 groups as compared to type 2 endometrial cancer group. Previous research also supports this notion and a threefold increased risk of endometrial cancer was found in diabetic women by Freiberg, Orsini, Mantzoros, & Wolk.^17^

Family history of malignancy was noted in 17(12.4%) patients with type I EC while 3(25%) patients with type II EC with P Value of 0.20. Family history is positively related with endometrial cancer as suggested in previous researches on significance of family history in predicating chances of endometrial cancers. Lucenteforte, et al. reported in their study that presence of endometrial cancer in first degree relatives is related to increased risk of endometrial cancer.^18^

In the past, researches have highlighted the protective effect of smoking by associating smoking with increased progesterone receptors (PGR) and homeobox A10 (HOXA10) expression in human endometrium and endometrial cells.^4^ In the current study, three patients (2.2%) out of 137 patients in type I endometrial group were smokers, while no smoking history was noted in type II endometrial group. Because of the social stigma attached to female smoking in Pakistani society, women are reluctant to disclose their smoking habit. As could be seen from the data, only three females from the entire sample reported smoking history. Therefore, no conclusive finding could be obtained regarding decreased risk of endometrial cancers due to smoking.

Polycystic Ovarian Syndrome and endometrial cancer are said to share common risk factors, but increased incidence of endometrial cancer in females with PCOs is not maintained decisively. A meta-analytic study that summarized findings of a previous study reported that PCO is not associated with increased risk of endometrial cancer.^19^ In the present study, only 2 Cases of type I endometrial cancer were reported to have PCO. Because very few cases were included of type II endometrial cancer, there is no case with diagnosed PCO in this group. It should be noted here that sample comprised of females of old age. As this is the age in which endometrial cancer is mostly diagnosed, and they might have undetected PCO in young age that remained undiagnosed due to Lack of awareness. Therefore, given the small number of reported cases of PCO in the present study, status of PCO as risk factor for endometrial cancer could not be established.

Hormone replacement therapy (HRT) has been associated with endometrial cancer in past studies in which longer duration of tamoxifen use was found to increase risk of endometrial cancer.^20^,^21^ In our sample only one patient with type II endometrial cancer had taken HRT while no patient with type I endometrial cancer reported having HRT in past. No significant finding could be obtained regarding risk value of HRT because of limited number of patients.

In type I EC group Adjuvant radiation was given to 47 patients and their mean survival time was found to be 113.68 months. Whereas in type II EC group 6 patients received Radiation and their mean survival time was found to be 24.72 months. Hence, present findings are consistent with past observations in which better survival time was reported for type I endometrial cancer.

### Limitations of the study

Limitations of this study are the retrospective type of study, small sample size, and mismatched sample distribution in the two groups. Moreover, because records were not maintained for the purpose of research, there was missing information related to few variables in standard record maintained by hospital. No conclusive finding could be obtained about certain risk factors because of small sample in one of the comparison groups that rendered statistical analysis insignificant.

## CONCLUSION

The results of this study suggest that the two types of endometrial cancer share many common etiologic factors. Despite the limitation of small numbers in one group this study confirms better survival in type 1 endometrial cancer.

## RECOMMENDATION

By **conducting similar studies with larger sample sizes,** balanced comparison group and interview-survey design, more accurate and generalized findings could be obtained pertaining to risk factors of endometrial cancers.
